# Inhibition of thrombin signaling after traumatic brain injury improves neurobehavioral dysfunction in 5xFAD mice

**DOI:** 10.1002/alz.091625

**Published:** 2025-01-03

**Authors:** Jaclyn Iannucci, Victoria Arismendi, Shreya Bandopadhyay, Paula Grammas, Lee A. Shapiro

**Affiliations:** ^1^ Texas A&M Health Science Center, Bryan, TX USA; ^2^ Roskamp Institute, Sarasota, FL USA

## Abstract

**Background:**

Traumatic brain injury (TBI) is a serious societal concern and is considered a major risk factor for the development of Alzheimer’s disease (AD) and related dementias. Identifying shared pathological mediators that contribute to the progression of AD following TBI may allow therapeutic targeting to reduce the likelihood of developing AD following TBI. Cerebrovascular dysfunction is present in both AD and TBI, and thrombin has been implicated as a mediator of cerebrovascular dysfunction and inflammation. Elevated thrombin levels and thrombin signaling following TBI have been associated with cognitive impairment. Thrombin has similarly been implicated as a pathological mediator in AD. Many of thrombin’s detrimental cellular effects have been linked to thrombin activation of protease activated receptors (PARs), including PAR‐1, which are expressed on a number of cell types in the brain and periphery, including endothelial cells, astrocytes, microglia, and neurons. Therefore, inhibiting thrombin activation of PAR‐1 may be a treatment strategy for AD pathogenesis after TBI. We hypothesize that targeting PAR‐1 activation will mitigate the TBI‐induced increase in AD pathogenesis.

**Methods:**

8‐week‐old male 5xFAD mice received either our lateral fluid percussion injury (FPI) model of TBI or sham surgery. This was followed 3 hours later by inhibition of PAR‐1 activation via treatment with a selective PAR‐1 inhibitor (SCH‐79797; 25µg/kg, i.p.) or vehicle control (DMSO). Behavioral measures included digigait assessment of acute motor deficits, depression‐associated behaviors, including social interaction and burrowing, and cognitive behaviors. Mice were subsequently sacrificed and tissue was collected for analysis of AD‐associated pathology as well as cerebrovascular dysfunction.

**Results:**

FPI causes chronic cerebrovascular alterations in 5xFAD mice. FPI was also found to induce deficits in depression‐associated behaviors in 5xFAD mice, most notably social interaction. FPI also induced hippocampal‐associated cognitive impairment in both the Y‐maze and Barnes maze. The impaired affective and cognitive behaviors were improved by SCH‐79797 treatment.

**Conclusions:**

These findings indicate that there is chronic cerebrovascular dysfunction in 5xFAD mice that can be exacerbated by FPI and highlight the potential for targeting thrombin and related signaling processes for mitigating these effects after injury.

